# Supplementary material for: Patterns of Intron Gain and Loss in Fungi
Source: PLoS Biol. 2004 Nov 30;2(12):e422. doi: 10.1371/journal.pbio.0020422 (PMC532390; doi:10.1371/journal.pbio.0020422)
Supplement: Table S1 — Also available at http://genes.mit.edu/NielsenEtAl/. (4.3 MB ZIP). [file pbio.0020422.st001.zip › NielsenEtAl/html/1283.html]

AN1913.1.NCU04020.1.MG04966.1.FG08761.1


```
 CLUSTAL W (1.82) Multiple Sequence Alignments - Introns Inserted


Sequence 1: AN1913.1	606 aa
Sequence 2: FG08761.1	610 aa
Sequence 3: NCU04020.1	618 aa
Sequence 4: MG04966.1	615 aa
Alignment Length: 621 aa
Number Identitical Residues: 320 aa
Alignment Score (without introns) 16071


MG04966.1 	MADSAASEP--PTDQVANLILDEVTGEK0VSKTELKRRQKARQKADEKAKKAAAAPPKPA
NCU04020.1	MADSSATTPAAAADQLANLHLDEVTGER~VSKSELKKRQKQRQKEAEKAAKAATAPPKAT
FG08761.1 	MAD--AAAPP--TEQVANLHLDEVTGEK~ISKTELKKRQKARQKEEEKKKKAAERGPAPA
AN1913.1  	MTDSNPVQEA--EASMANLLLDEVTGEK~VSKSELKRRQKLREKEAKKKEKEAAAPPKPA
          	*:*: .   .    .:*** *******: :**:***:*** *:*  :*  * *   * .:

MG04966.1 	AAK-KAAKPDEQ-ELNPN~QYFEIRSREVNGMLENPSGPNPYPHKFLVDYDPSQFDKDFK
NCU04020.1	SSKPKNAAGGEE-DLNPN~QYYEIRTRQVNELLKNPE-TNPYPHKFQVNYDDSNFVEEFG
FG08761.1 	PKKAAGGAEASEKDLTPN~QYFEIRSRNINKLRETKS-PNPYPHKFNVTYDLRKFVEEFG
AN1913.1  	AQKKVS-AEDEEANLTPN0QYFEIRSKRINKLRETKQ-PDPYPHKFQVTDDLRKYLKEYE
          	. *       .: :*.** **:***::.:* : :. . .:****** *  *  :: ::: 

MG04966.1 	HLKSGDVDKTREIRIAGRIFTKRSSGNKLIFYD~IKTGSDTTTTGSKMQIMCQAQYVKEG
NCU04020.1	SLKTGETLPEKELRIAGRIYNIRTAGSKLIFYD1IRTSADTKSIGTRMQVVCQAQLAKEG
FG08761.1 	HLKSGEHAKDKIIQIGARVHGKRASGAKLLFYD~VRT------EGVKVQIMCQAQEVREG
AN1913.1  	SLAKGEQKPDTTVRIAGRIYTKRSSGAKLIFYD~IRA------EGVKVQVVCQAQNA-SG
          	 * .*:      ::*..*:.  *::* **:*** :::       * ::*::**** . .*

MG04966.1 	GPTFEQQHEHLGRGDVIGIVGFPGR~TAPKAKLERGEEGELSLFATEVVQLSPSLHLLPG
NCU04020.1	GVPFEKQHAHLRRGDIIGIVGFPGR~TNPK-NLKEGEQGELSVFATEVQLLSPCLHMLPS
FG08761.1 	APSFEDQHEHLRRGDIIGIIGYPGR2TAPKTKIEKGEEGELSIFATEVVLLTPCLHALPD
AN1913.1  	AVSFEDQHEHLRRGDIVGIVGFPGR~SNPKNRPD----GELSIFATEVVLLAPCLHAIPS
          	. .**.** ** ***::**:*:*** : ** . .    ****:*****  *:*.** :*.

MG04966.1 	ERAGFTDGEKRFRMRYLDFMFNDKSREVLWQRSRIVKYIRDFFHDRRFIEVETPMMTSIA
NCU04020.1	VRYPFADAEQRARMRYLDMLWNDRSRETLWQRSRMVRYIRDFFHERRFIEVETPMMHAIA
FG08761.1 	DHYGFKDLEQRYRKRYLDLICNEKARNVFITRSKMITWIRKYFDERDFVEVETPMMNQIA
AN1913.1  	EHYGFQDKEQRYRQRYLDLIMNDKSRNVFITRSKMVTYIRNFFDQRDFVEVETPMMNAIA
          	 :  * * *:* * ****:: *:::*:.:  **::: :**.:*.:* *:*******  **

MG04966.1 	GGATALPFVTHHNEYDLDMFMRIAPELYLKMLVVGGYNKVFEIGKNFRNEGCDLTHNPEF
NCU04020.1	GGATALPFVTHHNDLDMDMFMRVAPELFLKKMIVGQFGKVFEMGKNFRNEGIDLTHNPEF
FG08761.1 	GGATAKPFTTHHNEYDMPMFMRVAPELYLKMLVVGGLNRVYEIGRQFRNEGADLTHNPEF
AN1913.1  	GGATAKPFITHHNEHDMNLFMRVAPELYLKMLIVGGLERVYELGRQFRNEGVDLTHNPEF
          	***** ** ****: *: :***:****:** ::**   :*:*:*::***** ********

MG04966.1 	TTIEAYAAYYDMYDVMDYTEELVSGLVKHLTGGYETKFTTQTGETYEVNWARPWKRVKIM
NCU04020.1	TSIEFYWAYADVYDLMSITEELVSSLVKELTGSYKTKFTNQHGETYEVNWEAPWRRVEMI
FG08761.1 	TTIEFYEAYADVNDLMKTTEEMISGLVKYLTGGYKTTFHTQSGEKYEVNWEAPWPRYEMI
AN1913.1  	TTCEFYWAYADVYDVMNLTEELVSGLVKHITGGYETTFHTQTGEEYKVNWKAPWRRVEMI
          	*: * * ** *: *:*. ***::*.*** :**.*:*.* .* ** *:***  ** * :::

MG04966.1 	PELERLTGKKFPPYSELHTDETNQFLRDLLKEKNIECTPPLTNARMLDKLIGEYLEETCI
NCU04020.1	PALEEATGEKFPPYDQLHTDETNAFLQRICKKMNVECPPPLTNARMIDKLTGEFIEETCI
FG08761.1 	PTLEEATGEKFPSGDQLHTQETNEFLKNVLKKMNLECTPPLTNARMIDKLVGEFIEEKCV
AN1913.1  	PALEEATGEKFPPGDQLHTAETGEFLKKVLKKTGVECSPPLTNARMLDKLVGEFIEETCI
          	* **. **:***. .:*** **. **: : *: .:**.********:*** **::**.*:

MG04966.1 	NPTFLMEHPQLMSPLAKYHRTEKGISERFEGFVCKKEICNAYTE0LNNPFDQRLRFEE0Q
NCU04020.1	NPTFILEHPQMMSPLAKYHRSKNGLCERFEAFVCKKEIANAYTE~LNNPFDQRLRFEE~Q
FG08761.1 	NPSFITGHPQVMSPLAKYHREIPGLCERFEAFVCKKEIANAYTE~LNDPFDQRLRFEE~Q
AN1913.1  	NPTFITGHPQMMSPLAKYHRQNAGLCERFEAFVCKKEIVNAYTE~LNDPFDQRLRFEE~Q
          	**:*:  ***:*********   *:.****.******* ***** **:********** *

MG04966.1 	ARQKAQGDDEAQMIDENFLRSLEYGL~PPTAGWGLGIDRLCMFLTNNATIREVLAFPFMR
NCU04020.1	ARQKDQGDDEAQLVDESFLNALEYGL1PPTGGWGLGIDRLAMFLTDNYSIREVLAFPFLR
FG08761.1 	ARQKDQGDDEAQLVDENFCTSLEYGL~PPTGGWGMGIDRMVMFLTDNYTIREVLTFPFMK
AN1913.1  	ARQKDQGDDEAQIIDENFCTSLEYGL~PPTGGWGMGIDRLVMFLTDNYSIKEVLAFPFMK
          	**** *******::**.*  :***** ***.***:****: ****:* :*:***:***::

MG04966.1 	DEKSG--PPVAAEVANVEPVPVEGIA1HK
NCU04020.1	EEKTAHKEKFAAEIAGVQPLPEEGIP1HK
FG08761.1 	DDKLEQKKQLAAEVAGIEPMPEEGIA1HK
AN1913.1  	DDKTAAEGKSAAEVVGIQPQPEEGIP1HK
          	::*   .   ***:..::* * ***. **
```
